# Admission patterns and clinical management of rare diseases in 46 pediatric intensive care units across China: a multicenter survey

**DOI:** 10.3389/fped.2026.1983050

**Published:** 2026-10-28

**Authors:** Dandan Chen, Guangfu Wen, Shanshan Xu, Qiuyue Zhang

**Affiliations:** 1 Key Laboratory of Reproductive Health Diseases Research and Translation of Ministry of Education, Department of Pediatrics, The First Affiliated Hospital, Hainan Medical University, Haikou, China; 2 Department of Pediatric Intensive Care Unit, Shengjing Hospital Affiliated to China Medical University, Shenyang, China; 3 Department of Pediatrics, The First Affiliated Hospital of Xiamen University, Xiamen, China

**Keywords:** China, cross-sectional, intensive care units, pediatric, rare diseases

## Abstract

**Objective:**

To investigate the admission status of pediatric patients with rare diseases in pediatric intensive care units (PICUs) across China, to provide an empirical basis for optimizing critical care management, resource allocation, and healthcare policy development for rare pediatric diseases.

**Methods:**

An online questionnaire survey was administered to gather cross-sectional data on the admission, diagnosis, treatment, and resource requirements of pediatric patients with rare diseases. A total of 65 questionnaires were returned from all invited member institutions of the Pediatric Critical Care Branch of the Chinese Medical Doctor Association across 30 provinces/municipalities; after exclusion of invalid questionnaires, 46 PICUs across 20 Chinese provincial-level regions constituted the final analytic sample. Data collection covered January 1, 2024, to December 31, 2024. Descriptive and comparative statistical analyses were performed on the valid data.

**Results:**

A total of 46 PICUs were included in the final analysis. In 2024, these PICUs admitted 1,765 pediatric patients with rare diseases, accounting for a median of 3.8% of PICU admissions across centers (range 2.1%–6.5%); the pooled proportion was 6.4% (2,231/34,625). The admitted cases covered 89 disorders (43.0%) listed in the *National Rare Disease Catalogs of China*. Inherited metabolic disorders (48.3%,95% CI: 45.2–51.4%) and neurological disorders (37.6%,95% CI: 34.6–40.6%) were the predominant disease categories, with children aged <3 years accounting for 43.7% of all cases. Most patients were admitted to the PICU due to status epilepticus, respiratory failure, and metabolic crises, indicating high demand for critical care interventions. The overall median time to diagnosis was 30 days; only 26.1% of centers were able to establish a diagnosis within 1 week. The clinical utilization rate of orphan drugs remained markedly low, with exorbitant medication costs and inadequate national health insurance coverage reported as the primary barriers. Healthcare institutions urgently identified the need for enhanced orphan drug accessibility, the establishment of dedicated diagnostic and assistance funds, and accelerated national insurance inclusion.

**Conclusion:**

Pediatric rare diseases frequently manifest during infancy and early childhood with acute, life-threatening presentations requiring PICU admission. Currently, clinical management faces substantial bottlenecks, including prolonged diagnostic delays and restricted accessibility to orphan drugs. National-level coordination is urgently required to construct rapid diagnostic green channels, strengthen medication support programs, and optimize the hierarchical healthcare delivery network.

## 1 Introduction

Rare diseases, also known as “orphan diseases,” represent a broad category of conditions with extremely low incidence or prevalence. They are characteristically heterogeneous and etiologically complex, often difficult to diagnose, lacking effective targeted therapeutics, and imposing substantial financial burdens. The clinical courses are frequently chronic, progressive, and debilitating, often leading to severe disability or premature death (1, 2) According to Orphanet statistics, more than 7,000 rare diseases have been cataloged to date (3). Although the prevalence of individual disorders is minimal, their collective global burden impacts an estimated 263–446 million individuals (3, 4). According to the *Comprehensive Report on Rare Diseases in China (2023)*, the population of patients with rare diseases in China has exceeded 20 million, with 69.9% diagnosed during childhood and requiring lifelong clinical management. China's healthcare system is organized into a tiered network of primary, secondary, and tertiary hospitals, with tertiary hospitals serving as referral centers for complex diseases. Rare disease diagnosis and treatment are concentrated in tertiary hospitals, particularly those in the national rare disease diagnosis and treatment collaboration network. In terms of insurance coverage, the National Rare Disease Catalog was first issued in 2018 and updated in 2023, now covering 207 conditions. As of 2024, approximately 100 orphan drugs have been included in the national medical insurance reimbursement list, although high out-of-pocket costs remain a barrier for many families. Critically ill pediatric patients with rare diseases frequently require admission to pediatric intensive care units (PICUs) for acute life-threatening complications, including respiratory failure, cardiac arrest, shock, and status epilepticus (5). However, existing investigations have primarily targeted broad hospital-wide patient populations or resident cohorts within single administrative regions, leaving critical care management and PICU admission patterns underreported (6–8). This study conducted a multicenter statistical analysis of rare disease admissions across 46 PICUs in China throughout a 1-year period (January 1, 2024, to December 31, 2024). We aimed to delineate admission profiles, disease spectra, diagnostic and therapeutic pathways, and operational challenges, providing an evidence base to guide critical care optimization, resource allocation, and health policy reform. This study aimed to: (1) describe the admission profiles and disease spectrum of pediatric rare diseases in PICUs across China; (2) assess diagnostic and therapeutic pathways, including time to diagnosis and orphan drug utilization; and (3) identify operational challenges and resource needs. Given the descriptive and exploratory nature of the study, no formal hypotheses were prespecified.

## 2 Materials and methods

### 2.1 Study population

A total of 65 questionnaires were returned from all invited member institutions of the Pediatric Critical Care Branch of the Chinese Medical Doctor Association across 30 provinces/municipalities. Pediatric patients admitted between January 1, 2024, and December 31, 2024, who met the diagnostic criteria defined in China's *First and Second National Catalogs of Rare Diseases* (9, 10), were included as the primary study cohort. After excluding duplicate submissions (*n* = 4), questionnaires with incomplete responses (*n* = 13), institutions without a PICU (*n* = 1), and centers that admitted no pediatric patients with rare diseases during the observation period (*n* = 1), the final analytical sample comprised 46 PICUs across 20 provincial-level regions. A confirmed diagnosis was defined as either (1) genetic confirmation by disease-specific testing, or (2) fulfillment of established clinical diagnostic criteria for the specific rare disease, or both. For diseases lacking genetic tests, clinical criteria alone were accepted. The year 2024 was selected because it was the most recent complete calendar year with full data availability across all participating centers and coincided with the implementation of the updated National Rare Disease Catalogs.

#### 2.1.1 Ethical approval

This study employed a retrospective observational design and did not collect identifiable personal health information or involve interventional procedures. The opening section of the questionnaire explicitly outlined the study objectives, data confidentiality commitments, and voluntary participation terms;because completing the questionnaire required active voluntary action by the designated physician, questionnaire submission was considered equivalent to informed consent. This approach was approved by the Ethics Committee of The First Affiliated Hospital of Hainan Medical University (Approval No.: 2026-KYL-076).

### 2.2 Study methods

#### 2.2.1 Questionnaire design and administration

The survey instrument was developed by the research team following a systematic literature review and expert panel consultations. Administered via an online survey platform, it was completed by designated senior intensive care physicians or PICU directors familiar with daily unit operations and rare disease workflows. The questions were distributed to the participating centers in advance, and the online questionnaire remained open for one month. The designated attending physicians completed the questionnaire by extracting the relevant data from electronic medical records rather than relying on recall.

#### 2.2.2 Questionnaire structure

The instrument comprised four structural modules:

- *Institutional baseline profile:* Institution name, accreditation level, hospital classification, PICU bed capacity, and annual total PICU admissions.
- *Rare disease admissions and demographics:* Volume and cumulative admission counts of cataloged and non-cataloged rare disease cases, broad disease classification, top five specific disease entities, primary indications for PICU admission, time to diagnosis, age distribution, proportions receiving invasive mechanical ventilation, continuous renal replacement therapy (CRRT), multidisciplinary team (MDT) consultations, and readmission rates.
- *Diagnostic and therapeutic challenges:* Core diagnostic modalities, primary diagnostic bottlenecks, orphan drug utilization rates, barriers to pharmacological therapy, and major therapeutic strategies.
- *Resource requirements and policy recommendations:* Institutional support priorities and structural health policy recommendations for national health administrative bodies.

### 2.3 Statistical analysis

Data processing and statistical analyses were performed using SPSS version 26.0 (IBM Corp., Armonk, NY, USA). Categorical variables are expressed as absolute counts (n) and percentages/proportions (%). Continuous variables are presented as medians [interquartile ranges (IQRs)]. Comparative analyses between institutional subgroups were performed using the Chi-square (*χ*^2^) test. Two-tailed *P*-values <0.05 were considered statistically significant. Patient-level analyses used 1,765 unique patients as the denominator, whereas admission-level analyses used 2,231 admission episodes. The pooled proportion of rare disease admissions was calculated at the admission level (2,231/34,625 = 6.4%). Center-level estimates (median and range across the 46 PICUs) are reported separately and are distinct from the pooled proportion.

## 3 Results

### 3.1 Baseline institutional characteristics

#### 3.1.1 Accreditation level and hospital classification

The 46 participating centers were distributed across 20 provincial-level administrative regions (covering Shanghai, Yunnan, Beijing, Jilin, Sichuan, Anhui, Guangdong, Guangxi, Jiangsu, Hebei, Henan, Zhejiang, Hainan, Hubei, Hunan, Gansu, Fujian, Guizhou, Chongqing, and Shaanxi), providing geographical representation across Eastern, Western, Southern, Northern, and Central China. Among the centers, 93.5% (*n* = 43) were tertiary class-A hospitals and 6.5% (*n* = 3) were tertiary class-B hospitals. By hospital type, general hospitals with pediatric departments accounted for 65.2% (*n* = 30), while specialized children's hospitals represented 34.8% (*n* = 16) (Figure 1).

**Figure 1:**
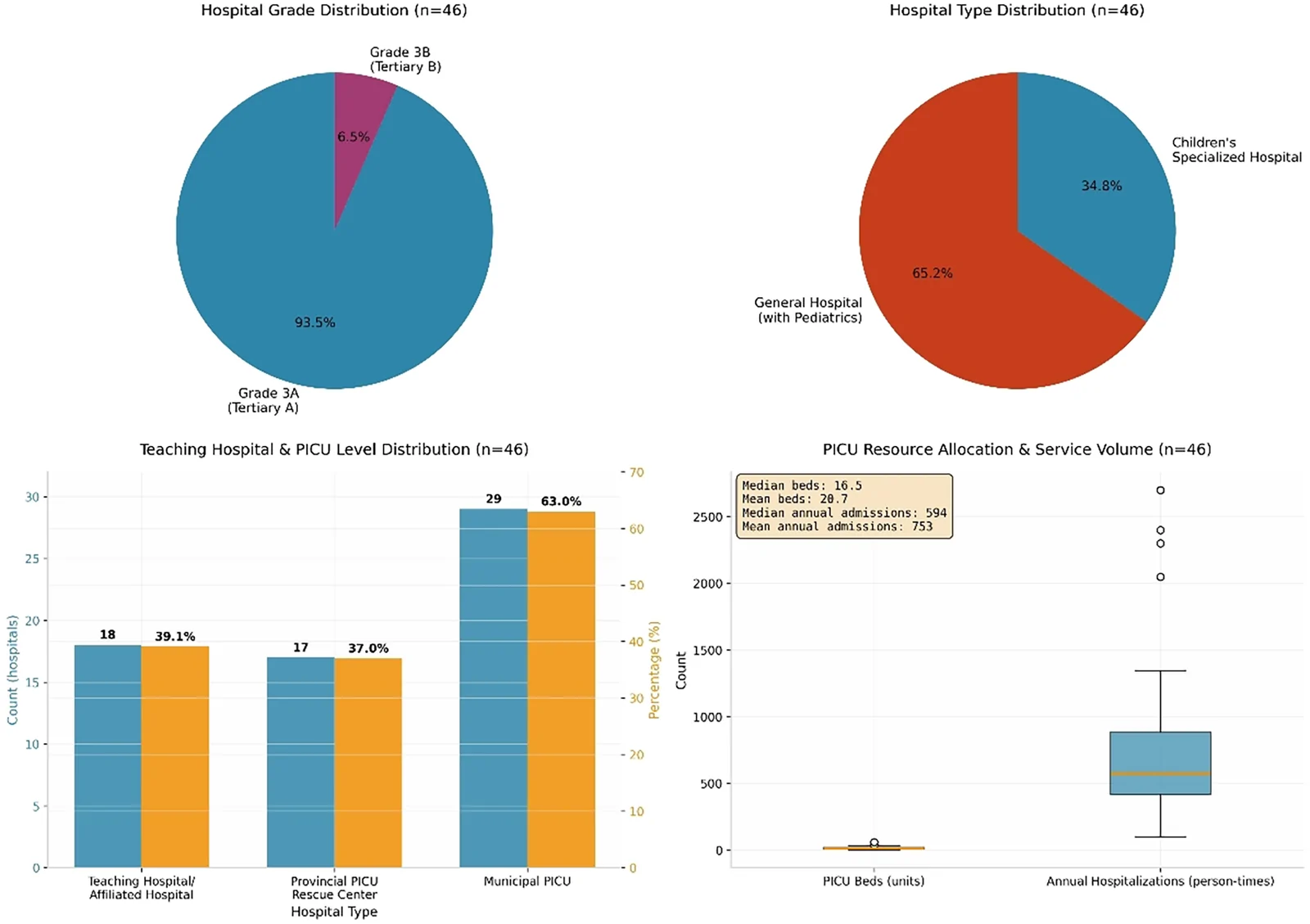
Analysis of baseline characteristics of 46 pediatric intensive care units in China.

#### 3.1.2 PICU resource allocation and service volume

- *Bed Capacity:* Across the 46 centers, the median PICU bed count was 16.5 (mean ± SD: 20.7 ± 14.2; range: 2–81; IQR: 10.0–25.8). Most units maintained between 10 and 26 beds, while a small subset of national medical centers exceeded 50 beds.
- *Clinical Service Volume:* In 2024, the 46 participating PICUs managed a cumulative total of 34,625 pediatric admissions. The median annual admission volume per center was 594 (mean ± SD: 753 ± 520; range: 20–2,715). Annual admissions were higher in provincial-level centers than in municipal-level institutions (median: 826 vs. 489 admissions).

### 3.2 Rare disease admission profiles

#### 3.2.1 Overall scale and case distribution

During 2024, the 46 centers admitted 1,765 uniquel pediatric patients with rare diseases, accounting for 2,231 cumulative admission episodes. Because some patients had more than one PICU admission during the study year, the number of admission episodes exceeded the number of unique patients. At the patient leve, 1,071 patients (60.7%) had a diagnosis listed in the *National Rare Disease Catalogs*, whereas 694 patients(39.3%) presented with clinical rare diseases not yet cataloged nationally. The 2,231 admission episodes included both first admissions and readmissions. The pooled proportion of rare disease admissions was 6.4% (2,231/34,625). Across centers, the per-center proportion ranged from 2.1% to 6.5%, with a median of 3.8%.The median volume of cataloged cases per PICU was 14 (range: 1–110), compared to a median of 3 non-cataloged cases (range: 0–337) (Figure 2).

**Figure 2:**
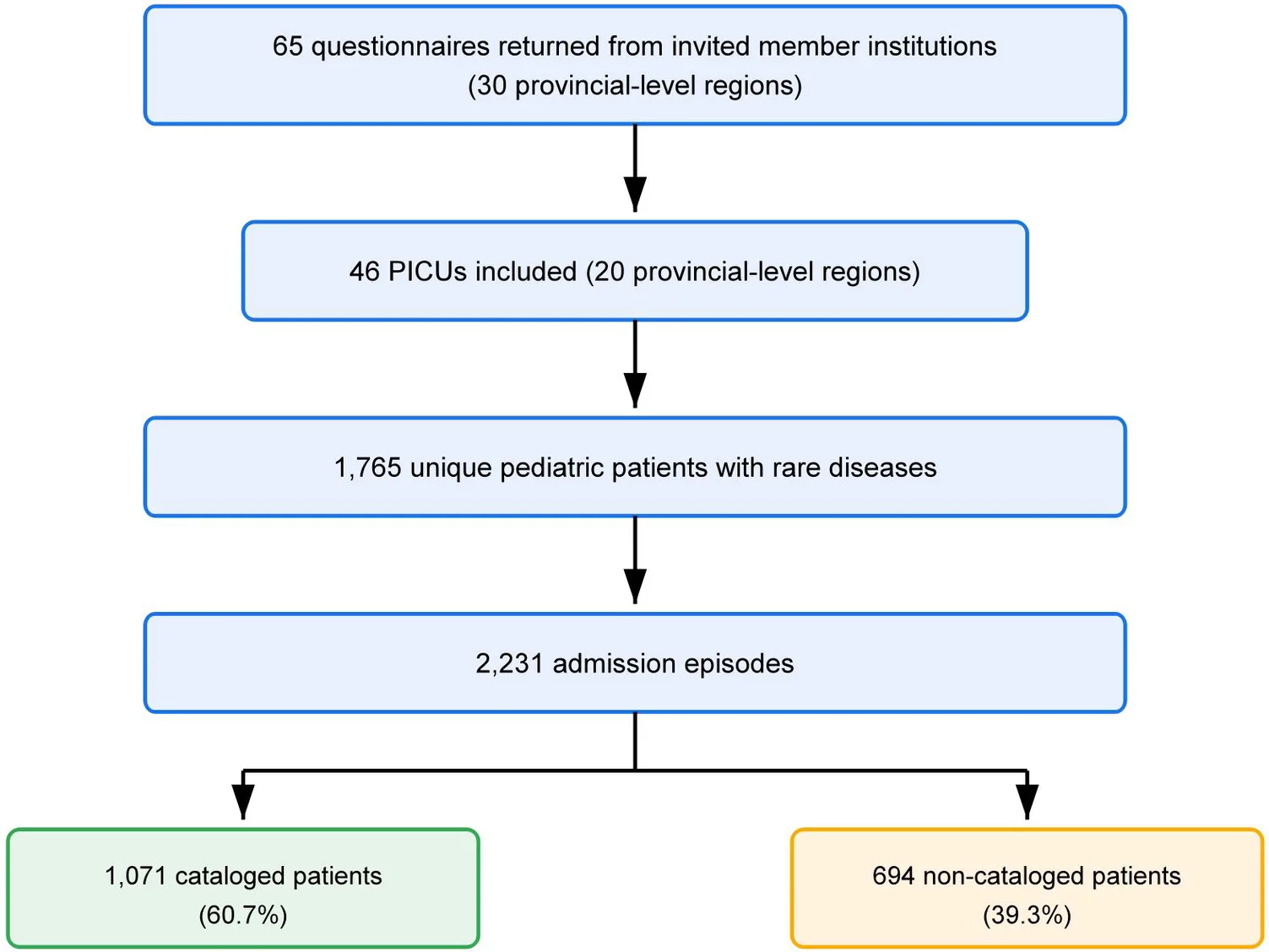
Flow diagram of the study population and rare disease admissions.

#### 3.2.2 Characteristics of cataloged diseases

Admitted cases spanned 89 distinct conditions listed in the national catalogs (53 from the First Batch and 36 from the Second Batch), representing 43.0% (89/207) of all cataloged conditions. Among the 1,071 cataloged rare disease patients, inherited metabolic disorders accounted for 48.3% (95% CI: 45.2–51.4%) and neurological disorders for 37.6% (95% CI: 34.6–40.6%). The remaining 14.1% included other disease categories such as endocrine, nutritional, and hematological disorders. Categories were assigned according to the classification used in the National Rare Disease Catalogs. The five most frequent conditions were:

- Autoimmune encephalitis: 23 episodes (11.3%)
- Spinal muscular atrophy (SMA): 17 episodes (8.3%)
- Infantile epileptic spasms syndrome: 10 episodes (4.9%)
- Neuroblastoma: 8 episodes (3.9%)
- Mitochondrial encephalomyopathy: 8 episodes (3.9%)

Together, these top five entities accounted for 32.3% of all cataloged rare disease admissions. The remaining 67.7% involved 84 additional rare diseases (including glycogen storage disease, methylmalonic acidemia, thalassemia major, congenital biliary atresia, hemophilia, atypical hemolytic uremic syndrome, hemophagocytic lymphohistiocytosis, congenital adrenal hyperplasia, Dravet syndrome, and primary immunodeficiency, among others); because of the large number of conditions, they were not listed individually.

### 3.3 Clinical presentations and management challenges

#### 3.3.1 Direct indications for PICU admission

The top three direct admission triggers were seizures or status epilepticus (*n* = 44, 93.5%), respiratory failure (*n* = 37, 80.4%), and severe metabolic crisis/decompensation (*n* = 35, 76.1%), collectively constituting 57.2% of primary presentations. Other indications included shock (*n* = 19, 41.3%), acute kidney injury/failure (*n* = 18, 39.1%), postoperative monitoring (*n* = 15, 32.6%), and other acute conditions such as severe anemia and sepsis (*n* = 2, 4.3%).

#### 3.3.2 Age distribution

Pediatric rare disease admissions were concentrated in early childhood:

- Infants (29 days to <1 year): 25.2%
- Toddlers (1 to <3 years): 18.5% (Children aged <3 years: 43.7%)
- Preschoolers (3 to <7 years): 20.4%
- School-aged children (7 to <13 years): 16.0%
- Adolescents (13 to <18 years): 4.5%

#### 3.3.3 Critical care interventions and service burden

- Invasive mechanical ventilation was required in a median of 20.6% of rare disease cases.
- Continuous renal replacement therapy (CRRT) was required in a median of 7.0% of cases.
- Interdisciplinary multidisciplinary team (MDT) management involving ≥3 subspecialties occurred in a median of 30.0% of cases.
- Readmission to the PICU (≥ 2 admissions/year) occurred in a median of 18.7% of rare disease patients(range: 0–60.0% across centers).

#### 3.3.4 Primary diagnostic modalities

Confirmatory diagnostic evidence relied on genetic testing (*n* = 46, 100.0%), biochemical/metabolic screening via blood and urine (*n* = 42, 91.3%), classic clinical phenotypes combined with family history (*n* = 38, 82.6%), and disease-specific antibody testing (*n* = 36, 78.3%). Genetic analysis, metabolic screening, and clinical/pedigree evaluation formed the core diagnostic triad (accounting for 68.5% of overall diagnostic confirmations). Genetic testing was used by all 46 institutions (100%), making it the only diagnostic modality employed universally across centers.

#### 3.3.5 Time to definitive diagnosis

Marked variation was observed in the time required to establish a diagnosis, with an overall median of approximately 30 days (ranging from 1 day to >1 year). More than half of the centers (54.3%) established diagnoses within 1–4 weeks, while only 26.1% achieved confirmation within 1 week (Table 1).

**Table 1:** Distribution of mean time to rare disease diagnosis across 46 PICUs.

Diagnostic Time Window	Number of PICUs (n)	Proportion (%, 95% CI)
≤7 days	12	26.1 (15.3–40.2)
8–30 days	25	54.3 (39.5–68.4)
> 30 days	9	19.6 (10.5–33.3)

Data are presented at the center level, with each participating PICU as the unit of analysis (*n* = 46). Each center reported the representative time required to establish a rare disease diagnosis in its PICU. Proportions are calculated using the 46 responding PICUs as the denominator and are presented as *n* (%) with 95% confidence intervals (Wilson method). These are center-level proportions and should be distinguished from patient-level or pooled proportions reported in the main text.

#### 3.3.6 Primary diagnostic bottlenecks

Key barriers to timely diagnosis in the PICU setting included prohibitive testing costs and family financial constraints (*n* = 38, 82.6%), unavailability of required assays in-house or extended turnaround times (*n* = 37, 76.1%), atypical or complex clinical presentations (*n* = 35, 76.1%), absence of rapid diagnostic pathways (*n* = 32, 69.6%), and clinician under-recognition of rare phenotypes (*n* = 28, 60.9%). Financial burden (22.4%), testing limitations (21.8%), and disease complexity (20.6%) accounted for 64.8% of reported diagnostic impediments.

#### 3.3.7 Orphan drug utilization and barriers

Among the 46 responding units, 35 centers (76.1%) reported that orphan drug utilization rates were <10% during PICU stays (Table 2).

**Table 2:** Barriers to orphan drug utilization across 46 PICUs (*n* = 46).

Identified Barriers to Orphan Drug Use in PICU	PICU count (n)	Proportion (%, 95% CI)
Exorbitant drug costs; absent or limited health insurance coverage	45	97.8 (88.7–99.6)
Complex procurement/application procedures (e.g., special approval pathways)	35	76.1 (61.9–86.3)
Lack of regulatory approval or domestic market unavailability	34	73.9 (59.5–84.6)
Insufficient clinical experience or lack of standardized guidelines	25	54.3 (39.5–68.4)
Critical clinical status unsuitable for drug administration, diagnostic delay, or difficulty assessing efficacy	18	39.1 (25.8–54.1)
Missed optimal therapeutic window	15	32.6 (20.6–47.2)

Data are presented as *n* (%) of responding PICUs; the denominator is 46 PICUs. Proportions are accompanied by 95% confidence intervals (Wilson method). These are center-level proportions and should be distinguished from patient-level or pooled proportions reported in the main text.

#### 3.3.8 Therapeutic interventions

The primary therapeutic modalities provided for rare diseases are summarized in Table 3.

**Table 3:** Primary therapeutic modalities provided for rare diseases across 46 PICUs (*n* = 46).

Primary therapeutic modalities	PICU count (*n*)	Proportion (%, 95% CI)
Disease-specific targeted therapies (e.g., enzyme replacement, diet modification, immunotherapy)	43	93.5 (82.2–97.7)
Symptomatic and life-support therapy for complications/critical illness (e.g., ventilation, CRRT, anti-infectives)	43	93.5 (82.2–97.7)
Surgical interventions (e.g., surgical resection)	24	52.2 (37.6–66.4)
Orphan drug pharmacotherapy	22	47.8 (33.6–62.4)

Data are presented as *n* (%) of responding PICUs; the denominator is 46 PICUs. Proportions are accompanied by 95% confidence intervals (Wilson method). These are center-level proportions and should be distinguished from patient-level or pooled proportions reported in the main text.

### 3.4 Resource demands and policy recommendations

Survey questions assessed institutional needs and national policy recommendations (100% response rate). Stratified analyses comparing general pediatric departments (*n* = 30) and children's hospitals (*n* = 16) showed no statistically significant differences across institutional needs or proposed policy priorities (*P* > 0.05) (Tables 4, 5).

**Table 4:** Comparison of institutional support needs between general and children's hospitals.

Institutional support needs	Overall cohort (*n* = 46)	General hospitals (*n* = 30)	Children's hospitals (*n* = 16)	*P*-value	Cramér's V
Enhancing orphan drug accessibility and affordability	42 (91.3%, 79.7–96.6)	27 (90.0%, 74.4–96.5)	15 (93.8%, 71.7–98.9)	>0.05	0.063
Establishing rapid diagnostic platforms/green channels	37 (80.4%,66.8–89.4)	24 (80.0%, 62.7–90.5)	13 (81.3%, 57.0–93.4)	>0.05	0.015
Strengthening multidisciplinary team (MDT) mechanisms	36 (78.3%,66.8–89.4)	23 (76.7%, 59.1–88.2)	13 (81.3%, 57.0–93.4)	>0.05	0.053
Enhancing specialized training in rare diseases	35 (76.1%,61.9–86.3)	23 (76.7%,59.1–88.2)	12 (75.0%, 50.5–89.8)	>0.05	0.019
Formulating clinical guidelines/expert consensus	28 (60.9%, 46.1–73.9)	18 (60.0%,42.3–75.4)	10 (62.5%, 38.6–81.5)	>0.05	0.025

Data are presented as *n* (%, 95% CI) of responding PICUs. *P*-values are from chi-square tests. Cramér's V is reported as a measure of effect size. These are center-level proportions and should be distinguished from patient-level or pooled proportions reported in the main text.

**Table 5:** Comparison of national-level priority support measures recommended by institutions.

National policy measures	Overall cohort (*n* = 46)	General hospitals (*n* = 30)	Children's hospitals (*n* = 16)	*χ*^2^ Value	*P*-value	Cramér's V
Establishing dedicated rare disease funds or medical assistance programs	42 (91.3%, 79.7–96.6)	28 (93.3%, 69.3–90.9)	14 (87.5%, 64.0–96.5)	0.605	>0.05	0.115
Accelerating orphan drug review/approval and national medical insurance inclusion	39 (84.8%, 71.8–92.4)	27 (90.0%, 74.4–96.5)	12 (75.0%, 50.5–89.8)	1.846	>0.05	0.200
Establishing national/region al diagnostic	38 (82.6%, 69.3–90.9)	26 (86.7%, 70.3–94.7)	12 (75.0%, 50.5–89.8)	1.232	>0.05	0.164
Supporting clinical research and national rare disease registries	34 (73.9%, 59.5–84.6)	23 (76.7%, 52.1–83.3)	11 (68.8%, 44.3–85.8)	0.396	>0.05	0.093
Disseminating standardized clinical pathways and management guidelines	32 (69.6%, 55.2–80.9)	21 (70.0%, 52.1–83.3)	11 (68.8%, 44.3–85.8)	0.012	>0.05	0.016

Data are presented as *n* (%, 95% CI) of responding PICUs. *P*-values are from chi-square tests. Cramér's V is reported as a measure of effect size. These are center-level proportions and should be distinguished from patient-level or pooled proportions reported in the main text.

## 4 Discussion

### 4.1 Core features and clinical significance of PICU rare disease admissions in China

Our findings demonstrate that the number of pediatric patients with rare diseases admitted to pediatric intensive care units (PICUs) in China is substantial. The 46 surveyed PICUs managed 1,765 rare disease cases across 2,231 admissions during 2024, with cataloged conditions representing 2.1% to 6.5% of total PICU admissions per center. This highlights the role of the PICU as a critical care epicenter for rare pediatric disorders.

Three key clinical characteristics define rare disease management in Chinese PICUs:

- *Predominance in early infancy:* Children aged <3 years comprised 43.7% of all rare disease admissions. This emphasizes that screening frameworks should concentrate heavily on neonates and young infants to facilitate early intervention and reduce mortality and secondary disability (11, 12).
- *Specific disease spectrum:* The disease spectrum centered heavily on inherited metabolic and neurological disorders, with metabolic entities occurring more frequently than reported in Western cohorts (13, 14). To avoid missed diagnoses, pediatric intensivists must maintain a high index of clinical suspicion for underlying rare conditions when managing acute metabolic decompensation or refractory status epilepticus.
- *High critical care dependency and recurrent admissions:* Patients exhibited significant need for invasive organ support (mechanical ventilation: 20.6%; CRRT: 7.0%) and had substantial readmission rates (18.7% readmitted ≥2 times/year). This pattern reflects disease chronicity with acute episodic exacerbations, demonstrating the necessity for coordinated care pathways that bridge PICUs and specialized outpatient services. The readmission rate varied widely across centers (0%–60.0%), likely reflecting differences in disease mix, severity, and follow-up infrastructure. Factors contributing to readmission may include recurrent metabolic crises, inadequate home care, and fragmented transition from PICU to subspecialty care. Additionally, genetic testing was utilized by 100% of surveyed centers, underscoring its role in definitive molecular diagnosis and targeted therapeutic decision-making in pediatric critical care. This is consistent with the research results both at home and abroad (9, 15, 16).

### 4.2 Key dilemmas in current clinical management

Our survey revealed three primary operational bottlenecks in PICU rare disease care:

- *Diagnostic Latency:* The median time to definitive diagnosis was approximately 30 days, with only 26.1% of institutions achieving confirmation within 1 week and 19.6% requiring more than 1 month (some exceeding 2 years). These findings mirror data from European cohorts reported by Faye et al. (15). Consistent with our findings, a recent Korean cohort of 101 pediatric patients with rare diseases reported that 52.5% received a diagnosis within 6 months, whereas 31.7% experienced a diagnostic odyssey exceeding one year; the number of hospitals visited, discordance between residence and diagnostic region, and age at symptom onset were significantly associated with longer time to diagnosis (17). The same Korean cohort further highlighted the family-level burden of rare diseases: 53.5% of families reported that a caregiver had stopped working to provide patient care, and 62.4% and 57.4% of families reported moderate-to-severe cognitive and motor limitations, respectively. These findings reinforce the need for integrated referral networks, structured MDT protocols, and psychosocial support for families, as recommended in our strategic priorities. Contributing factors include regional imbalances in advanced diagnostic infrastructure, high out-of-pocket costs for comprehensive genetic testing that lacks routine insurance coverage, and limited clinician familiarity with atypical presentations (9, 16, 18). A Singaporean cohort study of pediatric rare genetic diseases further showed that early use of next-generation sequencing substantially reduced the number of diagnostic tests and associated costs, supporting the argument for rapid diagnostic pathways in Asian healthcare systems (19). These factors may explain why the diagnostic delay observed in some Chinese cohorts appears longer than in European cohorts. In addition, cultural factors, including family decision-making patterns and health-seeking behavior, may influence diagnostic delays in China.
- *Limited Orphan Drug Accessibility:* A dual barrier exists, comprising drug unavailability and prohibitive costs Fully 76.1% of centers reported orphan drug utilization rates below 10%, consistent with findings by Heard et al. (13) and Park et al. (20) in Europe and the Middle East, confirming that restricted access to orphan drugs is a shared international challenge (21). In China, 73.9% of centers cited the lack of regulatory approval for specific orphan formulations—a rate higher than in Europe (13)—reflecting delays in translation, market authorization, and domestic availability. Furthermore, for approved agents, high out-of-pocket costs and incomplete basic medical insurance integration create financial barriers, contrasting with dedicated national rare disease funds in certain other national healthcare systems (14, 22).
- *Suboptimal Multidisciplinary and Hierarchical Coordination:* Rare diseases frequently involve multiorgan pathology requiring collaborative care across medical genetics, neurology, endocrinology, and rehabilitation. *Our survey assessed whether MDT consultations occurred rather than the quality of coordination; therefore, the finding that MDT was used in a median of 30.0% of cases should be interpreted as a measure of utilization rather than coordination quality.* Current PICU MDT practices often rely on ad-hoc, case-by-case consultations rather than structured protocols, limiting longitudinal care continuity. Furthermore, the referral pipeline between PICUs, subspecialty clinics, and community rehabilitation remains fragmented. The absence of an integrated continuum spanning acute stabilization, step-down transition, and chronic disease management** contributes to recurrent crises and repeat PICU admissions. This is in line with the conclusions of international studies such as Caillot C (23, 24). *In addition, the relationship between our survey findings and the policy recommendations is inferential rather than causal*.

### 4.3 Strategic recommendations for health system optimization

Based on our findings and China's clinical landscape, we propose the following strategic priorities, ranked by the proportion of centers endorsing each measure:

- Enhancing orphan drug accessibility and affordability (91.3%). National-level coordination is needed to expand insurance coverage, streamline approval, and establish a national rare disease fund. China has made progress (223 orphan drugs marketed; 136 reimbursed; 8.6 billion yuan allocated in 2024) (25), yet 97.8% of centers still report prohibitive costs and incomplete coverage, supporting a dedicated fund for ultra-high-cost drugs.
- Establishing rapid diagnostic platforms (80.4%). Provincial and municipal governments should create regional diagnostic hubs with rapid genetic testing capacity. Only 26.1% of centers diagnose within 1 week, and 82.6% cite prohibitive testing costs, supporting investment in rapid diagnostic infrastructure.
- Strengthening MDT mechanisms (78.3%). National and provincial authorities should standardize MDT protocols and referral pathways linking PICUs, subspecialty clinics, and community rehabilitation. The median MDT consultation rate was only 30.0%, supporting structured protocols.
- Enhancing specialized training (76.1%). National medical education authorities should incorporate rare disease recognition and acute crisis management into pediatric residency curricula, as 60.9% of centers identified clinician under-recognition as a diagnostic barrier.
- Formulating clinical guidelines (60.9%). National specialty societies should develop evidence-based guidelines for rare disease management in PICUs.

Some measures, such as accelerating orphan drug review and expanding insurance coverage, are already underway in China. Our data refine these efforts by identifying diagnostic delay and orphan drug inaccessibility as the most pressing bottlenecks.

### 4.4 Limitations and summary

This study represents a nationwide multicenter cross-sectional investigation characterizing the admission patterns and clinical demands of rare diseases across Chinese PICUs. Limitations include the single-year observation window (2024), which precludes longitudinal trend analysis. The single-year observation window (2024) may not capture annual or seasonal fluctuations in rare disease admissions, particularly for conditions with episodic or seasonal clustering. Future multi-year studies are needed to assess temporal trends and seasonal variation. In addition, detailed institutional characteristics were not available for all excluded centers, and therefore a formal comparison between included and excluded centers could not be performed. The National Rare Disease Catalogs do not include all rare diseases, and non-cataloged cases in our study may include genuine rare diseases that have not yet been included in the catalogs. This may have led to misclassification or underestimation of the true burden of rare diseases in PICUs. We did not collect data on which specific orphan drugs were available or unavailable at each center, nor whether utilization differed by disease type. This is a limitation of the survey design. Future studies should collect drug-level and disease-specific utilization data.

In summary, rare diseases represent a regular and resource-intensive component of pediatric intensive care in China, characterized by presentation in early childhood, high critical care dependency, and vulnerability to acute recurrence. Intensivists must maintain high clinical index of suspicion for early recognition. Addressing diagnostic delays, drug accessibility gaps, and coordination deficits will require coordinated health-policy reforms to improve long-term outcomes and quality of life for affected children.

## Data Availability

The original contributions presented in the study are included in the article/Supplementary Material, further inquiries can be directed to the corresponding authors.

## Appendix

1. The First Affiliated Hospital of Hainan Medical University
2. The First Dongguan Hospital Affiliated to Guangdong Medical University
3. The First Affiliated Hospital of Xiamen University
4. Children's Hospital of Soochow University
5. The Children's Hospital of Zhejiang University
6. West China Second Hospital, Sichuan University
7. Hunan Provincial People's Hospital
8. Hunan Children's Hospital
9. Affiliated Hospital of Zunyi Medical University
10. Children's Hospital Affiliated to Capital Institute of Pediatrics
11. Chengdu First People's Hospital
12. Guilin Maternal and Child Health Hospital, Guangxi
13. Jilin City Children's Hospital
14. The First Dongguan Hospital Affiliated to Guangdong Medical University
15. The First Affiliated Hospital of Kunming Medical University
16. Anhui Children's Hospital
17. The Second Hospital of Lanzhou University
18. Xinhua Hospital, Shanghai Jiao Tong University School of Medicine
19. Haikou People's Hospital, Hainan
20. Sanya Maternal and Child Health Hospital, Hainan
21. The Second Hospital of Hebei Medical University
22. Shanghai Children's Hospital，Affiliated to Shanghai Jiao Tong University School of Medicine
23. Henan Children's Hospital
24. Tongji Hospital, Tongji Medical College, Huazhong University of Science and Technology
25. The Second Affiliated Hospital of Wenzhou Medical University
26. The First Affiliated Hospital of Xinxiang Medical University
27. Nanjing Children's Hospital
28. The First Affiliated Hospital of Guangxi Medical University
29. Northwest Women's and Children's Hospital
30. Zhangzhou Municipal Hospital
31. The 900th Hospital of the Joint Logistics Support Force (PLA)
32. The First Hospital of Sanming
33. Gansu Maternal and Child Health Hospital
34. Yong'an General Hospital
35. The First Affiliated Hospital of Zhengzhou University
36. Taihe Hospital of Shiyan
37. Haikou Maternal and Child Health Hospital
38. The First People's Hospital of Tianmen
39. The Affiliated Hospital of North Sichuan Medical College
40. Yichang Central People's Hospital
41. Hainan Women and Children's Medical Center
42. Quanzhou First Hospital
43. Fujian Children's Hospital
44. Hainan General Hospital
45. Nanning First People's Hospital
46. Children's Hospital of Chongqing Medical University
